# Risk Mapping of Visceral Leishmaniasis: A Spatial Regression Model for Attica Region, Greece

**DOI:** 10.3390/tropicalmed3030083

**Published:** 2018-08-13

**Authors:** Polixeni Iliopoulou, Andreas Tsatsaris, Ioannis Katsios, Amalia Panagiotopoulou, Stelios Romaliades, Byron Papadopoulos, Yannis Tselentis

**Affiliations:** 1 School of Engineering, Department of Surveying and Geoinformatics Engineering, University Campus I, The University of West Attica, Egaleo, Athens 12243, Greece; atsats@uniwa.gr (A.T.); iokat@uniwa.gr (I.K.); apanagioto@gmail.com (A.P.); petrouchka03@yahoo.com (S.R.); 2 School of Medicine, The University of Crete, Iraklion 71003, Greece; vpapad@med.uoc.gr (B.P.); tselendi@med.uoc.gr (Y.T.)

**Keywords:** visceral leishmaniasis, Greece, geographic information systems, spatial regression

## Abstract

Visceral Leishmaniasis (VL) is endemic to the Attica region of Greece. The geographical distribution of VL cases was analyzed employing methods of spatial analysis in a GIS environment. A geographic database was constructed including data for the disease cases and environmental factors, such as land cover types, stray dog population, and socioeconomic factors. Classic and spatial regression models are presented that suggest the factors contributing most to the incidence of leishmaniasis are green urban areas and the population of stray dogs in the municipalities of Attica region. The results of the spatial regression model were more accurate, thus were used to produce a disease risk map. This map indicates the high-risk municipalities in which surveillance for the control of leishmaniasis is necessary.

## 1. Introduction

The purpose of this paper is to associate the geographical distribution of human cases of visceral leishmaniasis (VL) with several factors (environmental, socioeconomic and the population of stray dogs) which are considered to contribute to the spread of the disease. Data for the Attica region, Greece, are analyzed employing methods of spatial analysis in a GIS environment.

The Mediterranean region is one of the areas of the world where a high incidence of leishmaniasis is observed and it is recognized as a public health problem. In Greece, two types of Leishmaniasis occur: Visceral and Cutaneous. Visceral leishmaniasis (VL), known as kala-azar, is the most frequent type of the disease and occurs almost in the whole country. Phlebotomine sandflies (disease’s vector) are activated when the weather is warm (from May to October) and sting dogs and humans during the night. Dogs are the reservoir of *Leishmania infantum* which is the causative agent of VL [1,2].

Since the 1990s, Geographic Information Systems (GIS) and methods of Spatial Analysis have been extensively employed in the research concerning the geographical distribution of several diseases [3,4,5]. In addition, technologies, such as Global Positioning Systems (GPS) and remote sensing, allow the correlation of the geographical distribution of vector-borne diseases with environmental factors [6].

Concerning leishmaniasis, GIS techniques and regression models have been used in several studies to produce disease risk maps [6,7,8,9]. In addition, remote sensing approaches and spatial information technologies are used to monitor the main environmental factors related to the vectors of the disease such as rainfall, humidity, temperature, and vegetation [6,10,11].

Various environmental factors related to the spatial distribution of sandflies have been detected, such as meteorological factors, vegetation density, altitude, ecological disturbances, proximity to quarries and dumpsites, and the presence of animals hosting the disease and socioeconomic factors. These factors vary according to different environmental and socioeconomic conditions in different regions.

Meteorological factors, altitude, and vegetation are examined in several studies since sandflies appear in certain temperature, humidity, rainfall or elevation range, while certain types of vegetation seem to favor their prevalence [6,12,13]. Ecological disturbances such as deforestation, human movement, water control projects, road construction and the El Nino phenomenon are considered to influence the emergence and spread of zoonotic parasitic diseases including Leishmaniasis [2,14].

Apart from environmental factors, human activities increasing exposure to sandflies as well as the presence of animals, e.g., dogs, permissive to the leishmania life cycle, play critical roles in the development of VL. Socioeconomic conditions, daily activity, age, gender, ethnic and familial status are also associated with the disease [15]. Leishmaniases are also shown to be related to urbanization and migration of non-immune people to endemic areas [1,8].

In Greece, there is limited research on the geographical distribution of leishmaniasis. A study for the Greater Athens region in Greece investigated the main distribution patterns of the sandfly species responsible for the spread of VL [16]. A survey was carried out which involved sandfly collection in the period from May to October 1992, which is considered the season of activity of adult sandflies. The survey was accompanied by case studies, i.e., interviews with patients, to determine the way the disease was transmitted. The results gave indications that sandflies and patients were concentrated in the foothills of the mountains surrounding the Athens Basin, while the main environmental factors accounting for the spread of the disease were temperature, rainfall and the existence of quarries.

A subsequent study conducted in the same region concluded that the high distribution of sandflies in the vicinity of quarries can be explained by the fact that these sites are a mass of cracks, crevices and small or large caves which shelter considerable populations of rodents and—during the summer—stray dogs. Since dogs are the principal reservoirs of *L. infantum*, it seems that the quarries could be substantial foci for the spread of VL [17].

In this study, the main research questions concerned the relevance of several factors reported in the literature for predicting VL cases in Athens, Greece. More specifically, land cover types, socioeconomic characteristics and the population of stray dogs were examined as the main factors contributing to the spread of leishmaniasis. Certain land types such as quarries, dump sites and certain vegetation types are considered to favor the spread of the disease, since they tend to concentrate sandflies which are the disease’s vector. Poor socioeconomic conditions are considered to contribute to the concentration of VL cases, while stray dogs can be used to predict the VL cases because of their role as reservoirs in the leishmania life cycle. The choice of these factors relates to the literature, as presented above, but was also constrained by data availability. The analysis concerned a specific period when data on VL cases became available and was performed employing spatial units as observations, as opposed to examining individual patients.

## 2. Materials and Methods

**Figure 1:**
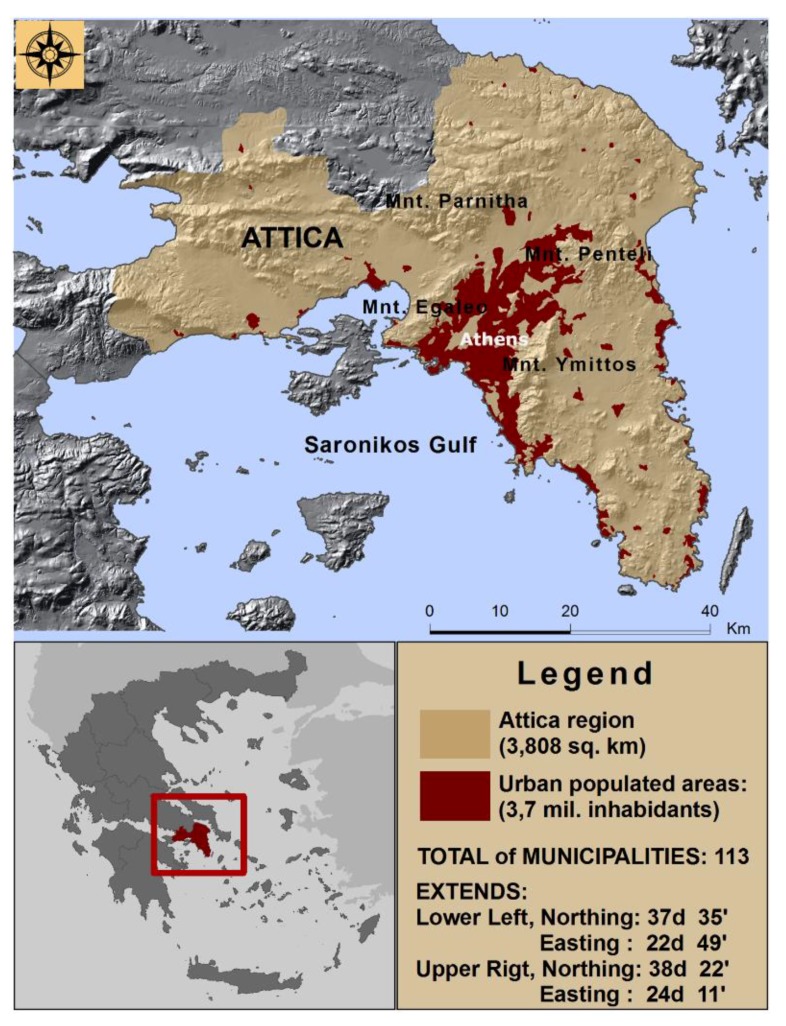
The study area (Source of Geographical Data: DCW 1:1M, ESRI^TM^).

In this paper, the study region is Attica in which Athens, the capital of Greece, is located (Figure 1). In Greece, VL is endemic to Attica which concentrates more than 50% of all cases in Greece. The study region has an area of 3808 square kilometers and a population of approximately 3.8 million inhabitants, while it comprises 113 municipalities.

Leishmaniasis cases in Greece have been recorded since 1961 by the Ministry of Health. Data include information on the age, sex and residence of the patients, the hospital of treatment and the type of leishmaniasis. Most reported cases concern VL and there is no indication as to whether they were fatal.

The dataset for this study consists of 1223 VL cases in Attica for the period 1961–2004. However, geographic reference, in terms of the municipality of the patient’s residence, is available for only 1121 cases. Since the exact address of the patients was available for a very limited number of cases, spatial analysis was carried out at the municipality level.

Several environmental factors related to the spread of VL, i.e., vegetation, the presence of quarries and dump sites, socioeconomic factors, and the population of stray dogs, were combined in a geographic database. For the design and construction of the geographic database, the platform of ArcGIS 9.3 (ESRI Redlands) was selected. Spatially referenced data which corresponded to the environmental factors influence the diffusion of the disease were used to form layers of information. Moreover, non-spatially referenced data (tabulated) were related to the above layers to expand the geographic database for the purposes of the research.

**Figure 2:**
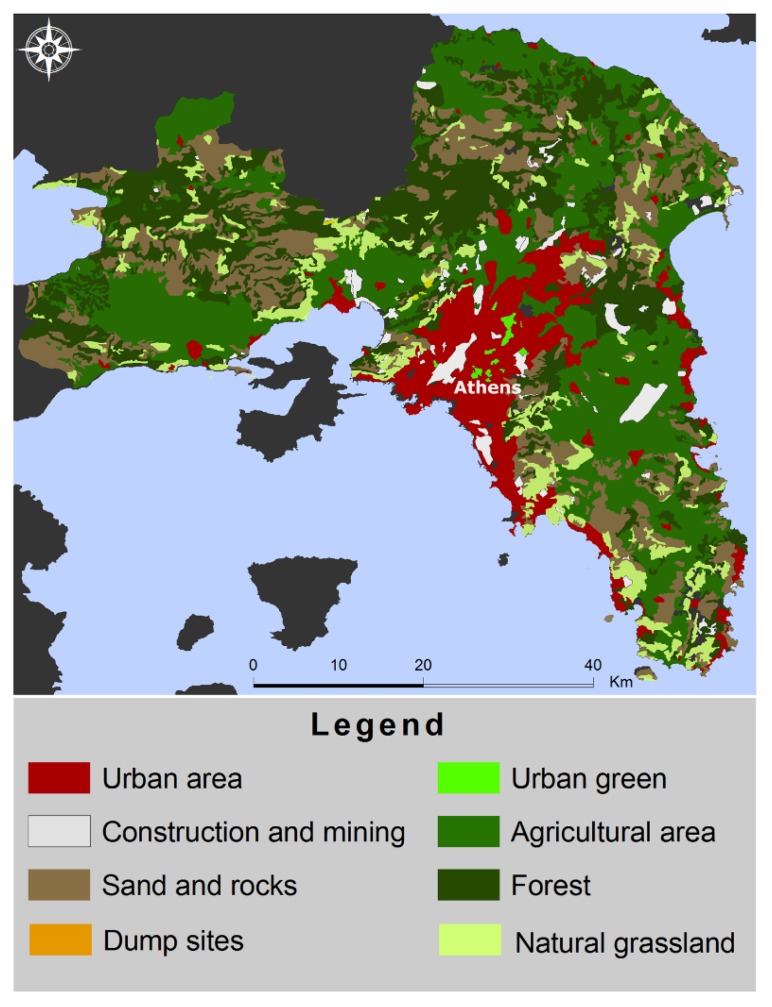
Polygons that represent the land cover according to the specifications of the project Corine Land Cover Greece 2000, funded by E.U., derived from LANDSAT TM.

In terms of the environmental factors, vegetation, quarries and dump sites were incorporated in the analysis employing the European Corine Land Cover data. Data from the European Corine Land Cover (CLC) 2000 were used, level 3 in particular, which identify 44 classes of land cover [18]. The area of each CLC category was measured for each municipality of Attica employing geo-processing techniques (Figure 2). Although data were available for all classes of land cover, four types of land cover are considered to have a relationship with the spread of leishmaniasis, i.e., dump sites, quarries, green urban areas and vineyards. Quarries and dumpsites were examined as CLC classes in that context.

Concerning the population of stray dogs, an estimation for 2005 was provided by the Ministry of Rural Development and Food.

Socioeconomic conditions in the municipalities of the Attica Region were analyzed in terms of indices expressing percentages of the population with poor living conditions or of dwellings with certain characteristics favoring the concentration of sandflies. Data for socioeconomic factors were obtained from the Statistical Service of Greece and include a variety of indices describing living conditions, structural characteristics of houses, socioeconomic structure and age structure.

Fifteen variables considered related to human VL cases were included in the analysis. They are four types of land cover, 10 variables describing housing conditions and the number of stray dogs. The list of variables (and their abbreviations) is presented in Table 1.

A final issue about data collection concerns their availability for different time periods. The initial dataset of VL cases concerned a forty-year period during which land cover has changed in Attica, while CLC data within this period are available for 1990 and 2000. Socioeconomic data were derived from the 2001 Population Census and the population of stray dogs was available only for 2005. Therefore, to match the time period of the rest of the data, only the VL cases for the period 1996–2004 were selected for spatial analysis. The total number of cases for this period is 246, out of which only 180 have a geographic reference, providing the municipality of patients’ residence.

Data were analyzed employing methods of correlation and regression analysis. In addition, descriptive statistics were calculated. Descriptive statistics and correlation analysis were performed with SPSS 25 statistical software.

At first, Pearson correlation coefficients were calculated between the number of VL cases and all explanatory variables. The variables with the strongest correlations were selected for regression analysis. A classic (OLS) regression model was calculated, employing the academic software GeoDa [19] which performs regression analysis employing map data (shapefiles). Since the analysis employs spatial data, it is recommended that the residuals of OLS should be examined for spatial autocorrelation. Spatial autocorrelation is a result of the continuity of geographic space, in the sense that variables are expected to have similar values in neighboring locations. Spatial autocorrelation violates the assumptions of the classical regression model, e.g., the randomness of residuals, and therefore the parameters of the model are misspecified. The most common way to measure spatial autocorrelation is the Moran’s index of spatial autocorrelation [20].

If spatial autocorrelation is detected, a spatial regression model would be more appropriate. The difference between the classic and the spatial regression model is that the latter considers spatial autocorrelation. In this study, the reason for employing a spatial regression model is that the frequency of VL cases is expected to be similar in neighboring municipalities since the environmental and socioeconomic factors are similar. For the calculation of the Moran’s index of spatial autocorrelation and the spatial regression model, a weights matrix is constructed indicating the number of neighbors for all the polygons in the GIS database which in this study are the municipalities of Attica. Two criteria are commonly used for the construction of the weights matrix from polygons: (a) the Rook criterion if neighbors share a common side; and (b) the Queen criterion if neighbors share a common side or vertex. In addition, different orders of these criteria may be selected if the neighbors of order 1 neighbors (which are the immediate neighbors) are included in the calculation of the weights matrix [19].

The spatial regression model was also produced using the GeoDa software [19]. Two of the most frequently used spatial regression models are available in GeoDa: the spatial lag and the spatial error models. In the first, a new independent variable enters the regression equation the values of which are estimated as the mean of the values of the dependent variable for all the neighboring municipalities. In the spatial error model, spatial dependence is handled through the error term; a spatially autoregressive error term is included in the equation:

y = Xβ + ε, with ε = λWε + u,(1)

where y is a vector of observations, W is a spatial weights matrix, X is a matrix of observations of the independent variables, ε is a vector of spatially autocorrelated error terms, u is a vector of independent and identically distributed errors and λ and β are parameters [19,21].

## 3. Results

**Figure 3:**
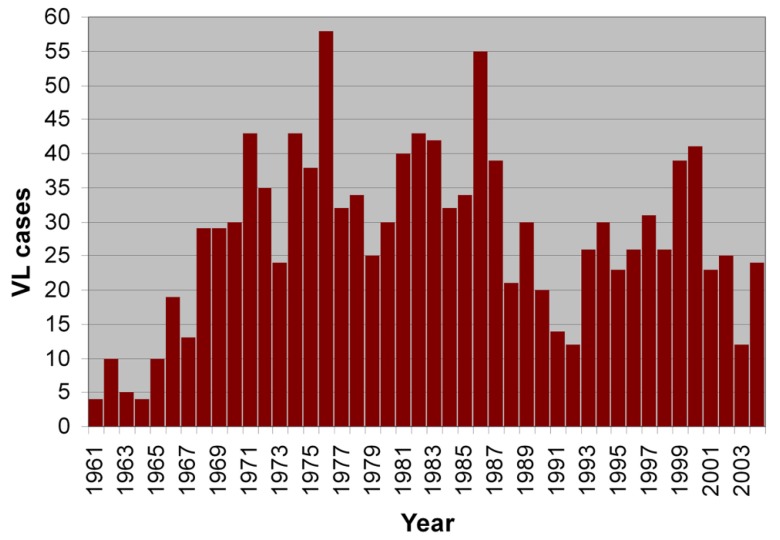
Temporal analysis of the VL cases for the period 1961–2004.

Temporal analysis of the 1223 VL cases for the period 1961–2004 in the study region indicated that the average is 30 cases per year, while some peaks were observed in 1976, 1986, 1993, 1994 and 2000 with approximately 50 cases per year (Figure 3). Consequently, the data present time periodicity, as has been reported in other studies [5].

**Figure 4:**
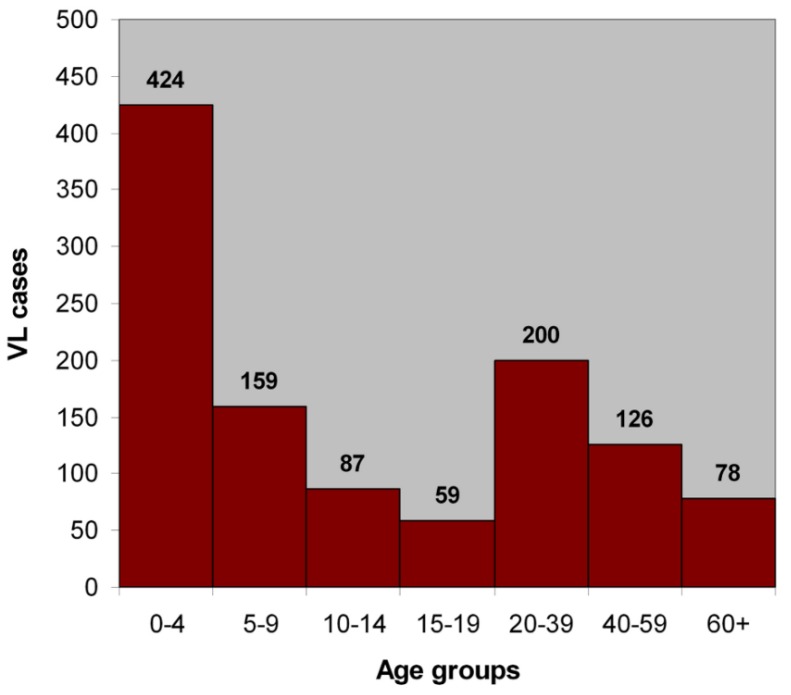
Age distribution of visceral leishmaniasis in Attica, Greece 1961–2004.

The results indicate that the disease has a higher effect on male patients (59%) which can be attributed to differences in daily activity. The age distribution showed that leishmaniasis affects mostly children, since more than 50% of the cases concern children under the age of 10 (Figure 4).

The correlation coefficients of VL cases with land cover types and stray dogs are shown in Table 2. Although all 44 categories of CLC-level 3 were tested, only those which might contribute to the spread of leishmaniasis are presented in Table 2.

Correlations of VL cases with socioeconomic factors are presented in Table 3.

In Table 2 and Table 3 significant linear relationships to the number VL cases were identified only for two variables: CLC-level 3 class “green urban areas” (Pearson r = 0.955) and the total number of stray dogs (Pearson r = 0.865). “Green urban areas” in CLC2000 are artificial, non-agricultural vegetated areas, i.e., areas with vegetation within the urban fabric such as parks created for recreational use, vegetated areas within the built area of the city, city squares and inner spaces of city blocks [18]. Therefore, green urban areas and the number of stray dogs were used as explanatory factors in a regression model for the prediction of VL cases in the municipalities of Attica.

The results of the classical regression model are shown in Table 4. The explanatory power of the classical regression model is very high (R^2^ = 0.918). In two separate simple regression models, R^2^ = 0.91 when the independent variable is URBAN_GREEN and R^2^ = 0.75 for the independent variable DOGS. Therefore, the variable which contributes most to the model is the green urban areas. The multicollinearity condition number in the OLS model is 4.15, i.e., less than 30, which is considered the threshold above which correlation among the independent variables is detected.

To test the residuals of OLS for spatial autocorrelation, a spatial weights matrix was created indicating the spatial neighbors for each municipality in the dataset. Different weights were tested and a Queen order 1 weights matrix was finally selected. The Queen criterion was adopted due to the irregular shape of the municipalities. In the weights matrix for each municipality, the number of neighboring municipalities is counted with the criterion being whether they share a common boundary or a vertex. The weights matrix is row standardized in GeoDa [19]. The weights matrix is employed for the calculation of the diagnostics for spatial dependence (Table 5) which are based on the analysis of the residuals of the OLS results of Table 4.

The Moran’s *I* statistic is highly significant suggesting spatial autocorrelation of the OLS residuals and therefore a spatial regression model is appropriate. Several trials of regression models, both OLS and spatial, were calculated using a variety of contiguity specifications for the weights matrix.

The selection of the appropriate model, i.e., a spatial lag or a spatial error regression model is based on the diagnostics for spatial dependence namely the Lagrange multipliers (LM) test statistics. Only the LM error statistic is significant, indicating that a spatial error regression model is selected [19]. The results of the spatial error model are shown in Table 6.

In addition to R square and significance level statistics, log likelihood, the Akaike Information Criterion (AIC) and the Schwarz Criterion (SC) were used to compare the fit of the regression models. The value of log likelihood has increased relative to OLS, while the values of AIC and SC have decreased, indicating an improvement of fit of the spatial regression model relative to OLS.

The coefficients of the explanatory variables are very similar for the two models. They are positive, indicating that the increase in the green urban areas and the increase in the population of stray dogs result in an increase of VL cases. All coefficients are significant, including the spatial autoregressive coefficient Lambda (λ) which is used for an improved estimation of the other coefficients. As measures of fit, the log likelihood, the Akaike information criterion, and the Schwarz criterion are used.

**Figure 5:**
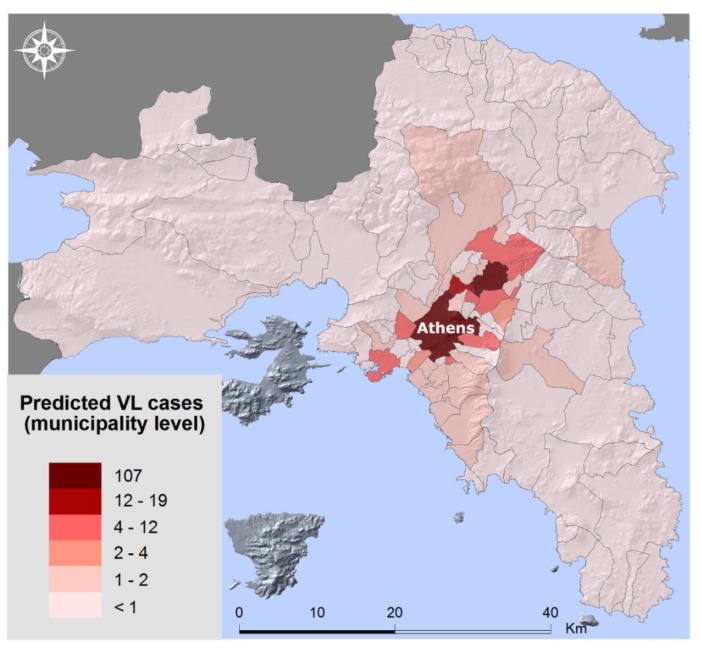
Risk map for leishmaniasis (spatial regression model).

Finally, a risk map for the disease of leishmaniasis was created (Figure 5) which shows the predicted VL cases as they were produced from the spatial error model. The areas with the higher risk for the occurrence of the disease are the center of the Greater Athens Region, i.e., the municipality of Athens, as well as some municipalities in the northern suburbs where urban green is rather extensive.

**Figure 6:**
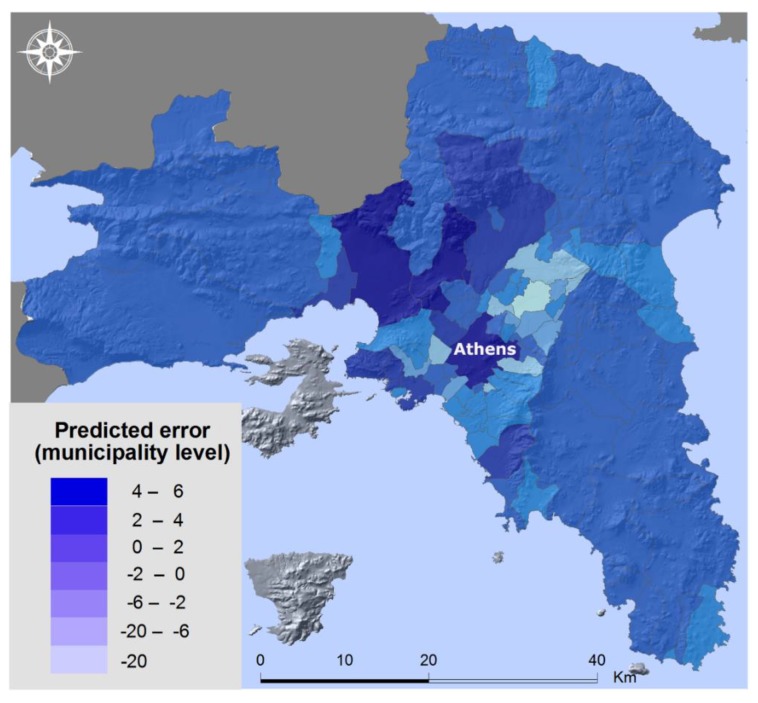
Prediction error (spatial regression model).

The prediction error of the regression model, i.e., the difference between observed and predicted values is presented in Figure 6. A positive difference represents an underestimation of VL cases by the model and this is observed mostly in the western suburbs of the Greater Athens Region.

Several versions of regression analysis were performed and the results were similar, even when VL cases for the whole period and not only for the years 1996–2004 were employed in the analysis.

## 4. Discussion

The parasite of leishmaniasis causes serious zoonotic infections, is endemic in the Attica region and, because it belongs to emerging zoonoses, is directly related to ecological and environmental changes, coupled with rapid population growth, domestic animals, and deforestation. The reemergence of old drug-resistant pathogens at the same time as evolutionary changes in hosts creates a more complicated scenario for treating these infections and significant public health implications at an increased cost of prevention measures.

Preventing the occurrence of zoonoses in humans is largely based on the treatment of animal diseases. Combating them is based on proper diagnosis, the implementation of hygiene and precautionary measures of the population and systematic surveillance for the early forecasting of epizootic diseases and epidemics.

Geographic Information Systems and spatial analysis work were exploited in the context of preventing the spread of leishmaniasis disease.

Thus, the purpose of this study was to describe and explain the geographical distribution of VL cases in the Attica region, Greece. A geographic database was constructed combining data of VL cases with a series of environmental and socioeconomic factors to examine the possible interplay among them, employing methods of spatial analysis. After several tests, a spatial regression model was selected to explain the geographical distribution of VL cases and produce a disease risk map employing VL cases for the period 1996–2004.

In that context, the factors which proved to contribute most to the geographical dispersion of leishmaniasis are the green urban areas and the number of stray dogs. The center of Athens concentrates most of the VL cases and includes extended parks and a large population of stray animals. In the northern suburbs, as well as some municipalities close to the city center, urban green is quite extensive and so is the presence of dogs (domesticated or not).

The rest of the land cover types did not prove to be significant for the prediction of the geographical distribution of the disease. The presence of dumps and quarries was examined in the context of CLC, i.e., by measuring the area covered by these CLC-level 3 classes in each municipality, and they did not prove significant for the geographical distribution of the disease. If data with geographic coordinates of VL cases were available, distances from dumps and quarries could be calculated. In that case, the association of VL cases with these land types might be established.

Similarly, correlation analysis of VL cases with socioeconomic factors did not result to significant correlation coefficients. Socioeconomic indices were produced from aggregate data at the municipality level and this is probably the reason that the association of socioeconomic factors with VL cases was not established. An analysis of individual VL cases would probably reveal the effect of socioeconomic conditions.

Two regression models were presented in this paper: classic and spatial regression models. Both models require only two independent variables: the green urban areas and the number of stray dogs in each municipality of the Attica Region. The first independent variable is changing slowly over time and is regularly measured through remote sensing techniques. The number of stray dogs is not easy to monitor in a reliable way. If these two variables are available, the prediction of the geographical distribution and the control of the disease will be greatly facilitated. In terms of urban green areas, measures for eliminating sandflies could be adopted, for example spraying. In terms of stray dogs, there are measures to control their population and provide appropriate care. Currently, these actions are left to animal welfare organizations.

Either the classic or the spatial regression model can produce reliable results concerning the prediction of VL cases in Attica, although the spatial regression model gives more accurate results. They are rather simple models and, according to the type of available data (tabular or spatial data), either model can be selected. The resulting disease risk maps can be used to establish a surveillance system for the control of the disease. The prediction error was presented for the spatial regression model and it shows an underestimation of VL cases in the western suburbs of Attica where the social conditions are considered less favorable and the major dump sites of the Greater Athens Region are located, attracting sandflies. It is possible that different socioeconomic data at a more detailed spatial level could further improve the spatial prediction of VL cases.

The possible limitations of the model relate to data availability and the scale of analysis. Since the data of VL cases concern a specific period (1996–2004), there was an attempt to relate them to land use types, socioeconomic characteristics and stray dogs of approximately the same time. However, there are additional data limitations. Data on VL cases did not include the exact address of the patients for most cases, but only the municipality of residence. If the exact address were available, the analysis would be conducted with points, and not areas, as observations, and the results might be different. Land cover types, as reported by the European Corine Land Cover, are not detailed enough and the same is true for the socioeconomic characteristics, which are averages at the municipality level. In that respect, factors which are reported in the literature as having impact on individual VL cases, i.e., the surrounding land use or the specific socioeconomic conditions of individual patients, may not prove significant at a regional scale.

In terms of future research, alternative statistical models could be used, such as a spatial Poisson regression model. Given the nature of VL cases data, i.e., discrete values and rare incidence, this would be an appropriate model but with different software requirements in comparison to this study.

## Figures and Tables

**Table 1:** Explanatory variables for human VL cases.

1	The area of mineral extraction sites
2	The area of dump sites
3	The area of green urban areas (URBAN_GREEN)
4	The area of vineyards (in square kilometers)
5	The number of stray dogs (DOGS)
6	Percent of population between 0–14 years (CHILDREN)
7	Percent of population with lower socioeconomic status (LOWER STATUS)
8	Number of family members in a dwelling (FAMILY MEMBERS)
9	Number of persons per room of dwelling (PERSONS PER ROOM)
10	Existence of sewage in the house (SEWAGE)
11	Existence of central heating (CENTRAL HEATING)
12	Percent of one- or two-story houses (ONE STORY HOUSES)
13	Percent of old buildings (before 1945) (OLD BUILDINGS)
14	Percent of buildings made of stone (STONE)
15	Percent of buildings with roof covered with tiles (TILES)

**Table 2:** Correlations (land cover and stray dogs).

		Mineral Extraction Sites (sq. km.)	Dump Sites (sq. km.)	Green Urban Areas (sq. km)	Vineyards (sq. km.)	Stray Dogs (Thousands)
VL cases 1996–2004	Pearson Correlation	−0.018	0.031	0.955	−0.024	0.865
	Sig. (2-tailed)	0.853	0.746	0.000	0.802	0.000

**Table 3:** Correlations (socioeconomic characteristics).

VL Cases 1996–2004	Pearson Correlation	Sig. (2-Tailed)
Children	−0.204	0.092
Lower Status	−0.046	0.709
Family Members	−0.036	0.767
Persons Per Room	−0.060	0.626
Sewage	0.058	0.637
Central Heating	0.121	0.321
One Story Houses	−0.214	0.084
Old Buildings	0.182	0.144
Stone	0.074	0.557
Tiles	−0.055	0.664

**Table 4:** Ordinary Least Squares Estimation.

Variable	Coefficient	Std. Error	*t*-Statistics	Probability
CONSTANT	−0.6880	0.3301	−2.0843	0.0394
URBAN_GREEN	27.4660	1.7506	15.6944	0.0000
DOGS	0.3183	0.1013	3.1438	0.0021
Adjusted R^2^	0.918			
Log likelihood	−293.061			
Multicollinearity condition number	4.1586			
Log likelihood	−293.061			
Akaike info criterion	592.122			
Schwarz criterion	600.434			

**Table 5:** Diagnostics for spatial dependence.

Test	MI/DF	Value	Probability
Moran’s *I* (error)	0.2200	3.9088	0.0001
Lagrange Multiplier (lag)	1	2.6999	0.1004
Lagrange Multiplier (error)	1	13.1514	0.0003

**Table 6:** Spatial Error Model.

Variable	Coefficient	Std. Error	*z*-Value	Probability
CONSTANT	−0.9372	0.4532	−2.0681	0.0386
URBAN_GREEN	27.4502	1.5959	17.2006	0.0000
DOGS	0.3891	0.0940	4.1399	0.0000
LAMBDA	0.4127	0.1105	3.7335	0.0002
R^2^	0.9298			
Log likelihood	−287.157			
Akaike info criterion	580.314			
Schwarz criterion	588.626			
